# The efficacy of new drug regimens in treating newly diagnosed high-risk cytogenetic multiple myeloma patients: a systematic literature review and meta-analysis

**DOI:** 10.3389/fmed.2025.1575914

**Published:** 2025-05-13

**Authors:** Huixing Zhou, Wenming Chen

**Affiliations:** Department of Hematology, Beijing Chao-Yang Hospital, Capital Medical University, Multiple Myeloma Research Center of Beijing, Beijing, China

**Keywords:** multiple myeloma, high-risk cytogenetics, novel drug regimens, CD38 antibodies, progression-free survival, minimal residual disease, systematic review, meta-analysis

## Abstract

**Introduction:**

Multiple myeloma (MM) is a plasma cell malignancy comprising 10% of hematologic cancers, associated with bone marrow dysfunction and organ damage. High-risk cytogenetic MM patients, identified by specific genetic abnormalities, face poor outcomes despite recent advancements. Traditional treatments often prove inadequate, necessitating novel regimens. This review assesses the efficacy of emerging therapies—next-generation proteasome inhibitors, immunomodulatory drugs, and CD38-targeting agents—aimed at improving outcomes for this patient subset.

**Methods:**

A systematic review and meta-analysis were performed, analyzing data from 18 randomized controlled trials (RCTs) involving high-risk MM patients treated with new drug combinations. Data extraction, quality assessment, and meta-analysis were conducted using a Bayesian fixed-effects model.

**Results:**

For transplant-eligible patients, CD38-based therapies reduced progression or death risk by 33% during induction and 48% during maintenance. They improved progression-free survival (PFS) by 38% in induction and 57% in maintenance and increased minimal residual disease (MRD) negativity by 38%. Dual novel drug regimens also enhanced MRD negativity, but Elotuzumab and Ixazomib regimens showed limited impact. Carfilzomib-based therapies showed varying PFS and survival benefits.

**Conclusion:**

CD38-targeted regimens notably improve outcomes in high-risk cytogenetic MM, especially for transplant-eligible patients, by reducing disease progression, enhancing PFS, and increasing MRD negativity. Dual novel regimens show promise in MRD improvements. These findings support the potential of tailored therapeutic strategies to optimize patient care.

## 1 Introduction

Multiple myeloma (MM) is a malignant hematological disorder characterized by the proliferation of abnormal plasma cells in the bone marrow, accounting for about 10% of all hematologic cancers (1). This disease leads to impaired marrow function, accumulation of abnormal protein (M protein) in the blood, and related organ dysfunction (2). Despite significant advancements in the treatment of MM, particularly with the introduction of new drugs and therapies, the outcomes for newly diagnosed patients with high-risk cytogenetic features remain suboptimal. The International Myeloma Working Group (IMWG) has updated the definition of high-risk multiple myeloma based on certain cytogenetic abnormalities (3). High-risk cytogenetic abnormalities, such as translocations t(4;14), t(14;16), t(14;20), deletions del(17p), and gain or amplification of chromosome 1q(1q+), are associated with poor prognosis and shorter survival, raising the bar for therapeutic strategies and efficacy assessment (4). Approximately 15%–20% of newly diagnosed MM (NDMM) patients and up to 30% of relapsed or refractory MM (RRMM) patients are classified as high-risk (5, 6).

Traditional treatment modalities, including chemotherapy, radiotherapy, and stem cell transplantation, are effective for patients with standard risk MM but often fail to provide satisfactory long-term survival for those with high-risk cytogenetic profiles (7). Therefore, developing new drug regimens tailored to this specific patient population has become a focal point of clinical research. The emergence of various new drugs and treatment strategies, such as next-generation proteasome inhibitors (Carfilzomib, Ixazomib), immunomodulatory drugs (Pomalidomide), CD38 antibodies (Daratumumab, Isatuximab), and the Elotuzumab monoclonal antibody, offers new hope for treating high-risk MM (HRMM) patients. These new agents, with their unique mechanisms of action, aim to improve treatment outcomes, extend progression-free survival (PFS) and overall survival (OS), and reduce the incidence of minimal residual disease (MRD).

This systematic literature review and meta-analysis aims to assess the efficacy of these new drug regimens in treating newly diagnosed patients with high-risk cytogenetic MM. By analyzing existing clinical study data in depth, this work aims to provide scientific evidence for clinicians that may help to guide the selection of new drug regimens, tailor strategies for high-risk NDMM patients, and ultimately enhance treatment outcomes and quality of life for this specific patient group.

## 2 Methods

This systemic literature review (SLR) and meta-analysis followed the Preferred Reporting Items for Systematic Review and Meta-analyses (PRISMA 2020) extension statement (8).

### 2.1 Search strategy and selection criteria

Three English electronic databases (PubMed, Embase and Cochrane) were searched on August 8, 2022 and updated on October 24, 2023. Our search strategies are reported in detail (Supplementary Appendix 1). Our search result was limited to English-language studies. Cytogenetically defined HRMM was defined as the presence of t(4;14), t(14;16), t(14;20), del(17p) or gain or amplification 1q(1q+). Inclusion criteria were: (1) NDMM patients with high-risk cytogenetic features; (2) treatment regimens incorporating newly approved drugs, irrespective of whether these are used in monotherapy or in combination with other drugs, without restrictions on dosage and frequency: Carfilzomib, Ixazomib, Pomalidomide, Daratumumab, Isatuximab, Elotuzumab; (3) outcome measures included MRD, PFS, OS, all-cause mortality; the number of disease progression or death; (4) Only randomized controlled trials (RCTs) were included. Exclusion criteria were: (1) patients with relapsed or refractory MM or without high-risk cytogenetic features; (2) treatment regimens did not incorporate new drugs; (3) studies that did not report relevant outcome measures; (4) non-RCTs.

### 2.2 Study selection

Two reviewers independently screened the titles and abstracts of all records retrieved from databases on EndNote to identify studies that appeared to meet the inclusion criteria. The full reports of all potentially eligible studies were obtained and assessed independently by the two reviewers to determine whether to include or exclude them. Any disagreements between the reviewers were resolved through discussion or with the assistance of the third reviewer.

### 2.3 Data extraction

Data extraction was independently performed by two reviewers using a specifically designed data extraction form, including study characteristics (i.e., country, type of study, NCT number, phase of study, center), participant characteristics (i.e., sample size, age, and definition of high risk), treatment regimen characteristics (i.e., phase of treatment, transplant status, treatment protocols, and number of cycles), and outcomes. In cases of disagreement between the reviewers, resolution was achieved through discussion or with the assistance of an additional reviewer.

### 2.4 Assessment of risk of bias

Quality assessment was done using the revised Cochrane Risk of Bias Tool for randomized trials (RoB 1.0) (9). Two independent authors assessed the quality of eligible RCTs according to the Cochrane Risk of Bias Tool (RoB 1.0), which includes sequence generation, allocation concealment, blinding of participants and personnel, blinding of outcome assessment, incomplete outcome data, and selective outcome reporting (9). Any disagreements were resolved by discussion with assistance from a third party when necessary.

### 2.5 Data analysis

The meta-analysis was conducted using the Bayesian fixed-effects model in Review Manager 5.4, to present direct comparison results between treatment interventions. For dichotomous variables, risk ratios (RRs) and their respective 95% confidence intervals (CIs) were estimated. For continuous variables, hazard ratios (HRs) and their 95% CIs were estimated when possible. Pooling of data for meta-analysis was performed when two or more studies with clinical and methodological homogeneity provided applicable results. A fixed-effects model was used for meta-analyses. We defined *I^2^* ≥ 50% with a statistically significant *Q* test result (*p* < 0.1) as evidence of substantial levels of heterogeneity (10). A funnel plot used for assessing publication bias was not performed due to the insufficient number of studies for each outcome (i.e., less than 10 studies). When there is insufficient data to conduct a meta-analysis, we perform descriptive analysis on the data.

## 3 Results

### 3.1 Results of study selection

**Figure 1:**
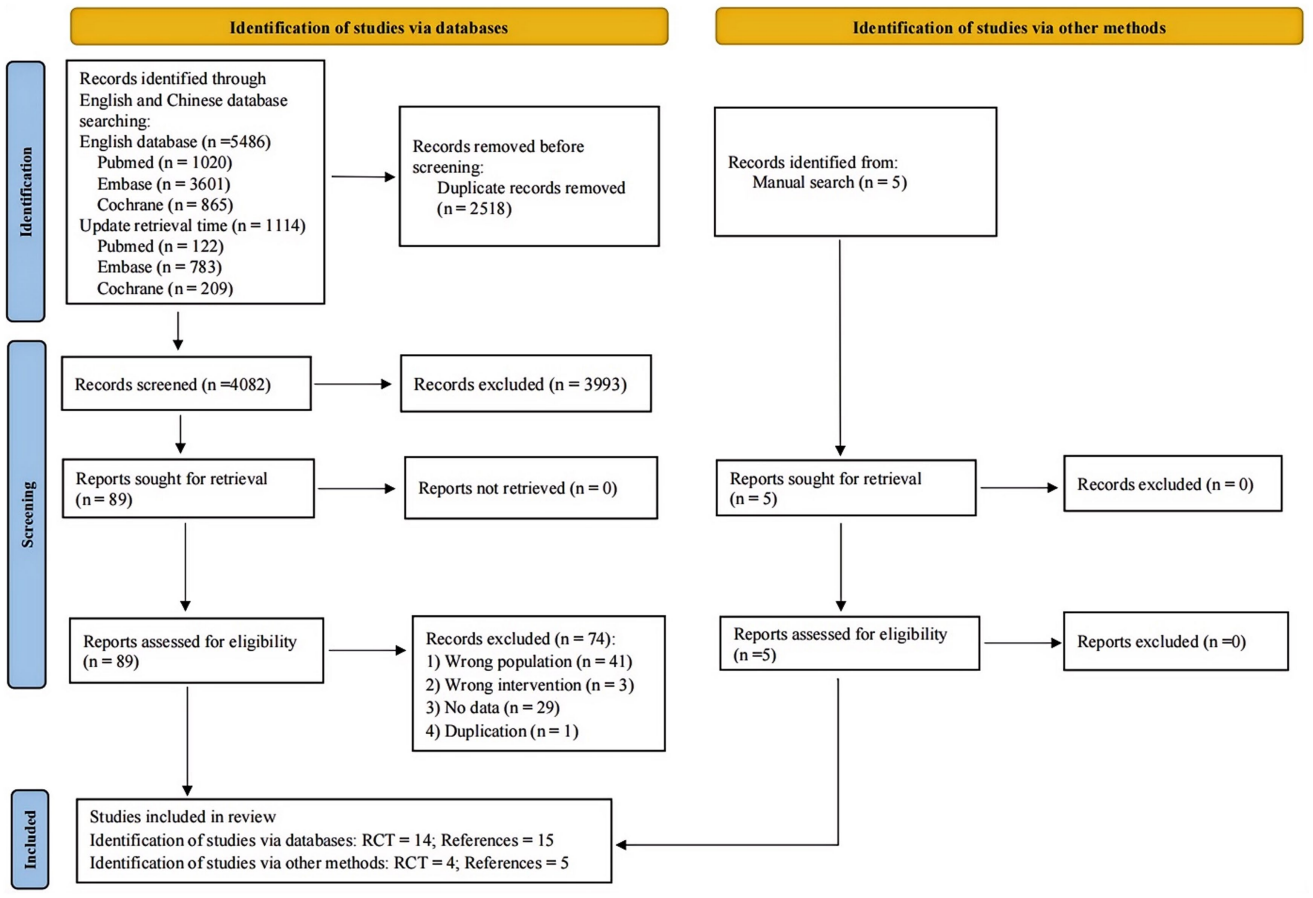
PRISMA flow diagram.

A total of 6,600 articles were retrieved from three English databases (PubMed, Embase and Cochrane) and 5 additional articles were obtained from other sources. After deduplication, 4,084 articles were screened initially. Eighty-nine articles were included for full-text screening after the initial screening. Following the screening, 18 randomized controlled trials (reported in 20 papers) were included, of which 2 RCTs (reported in 4 papers) came from other sources. The baseline characteristics and treatment measures of the included studies are detailed in Supplementary Tables S1, S2. The quality assessment included 18 studies. All studies exhibited low risk of bias in random sequence generation, indicating proper randomization and blinding. Blinding of outcome assessment was generally maintained well, ensuring objective measurements. However, several studies showed high risk of bias in blinding participants and personnel. A few studies had unclear risks in allocation concealment and selective reporting due to insufficient methodological details. The results of quality assessment are shown in Supplementary Figure S1. The procedure of study selection was presented in the PRISMA flow diagram (Figure 1).

### 3.2 For transplant eligible patients

#### 3.2.1 CD38-based regimens

**Figure 2:**
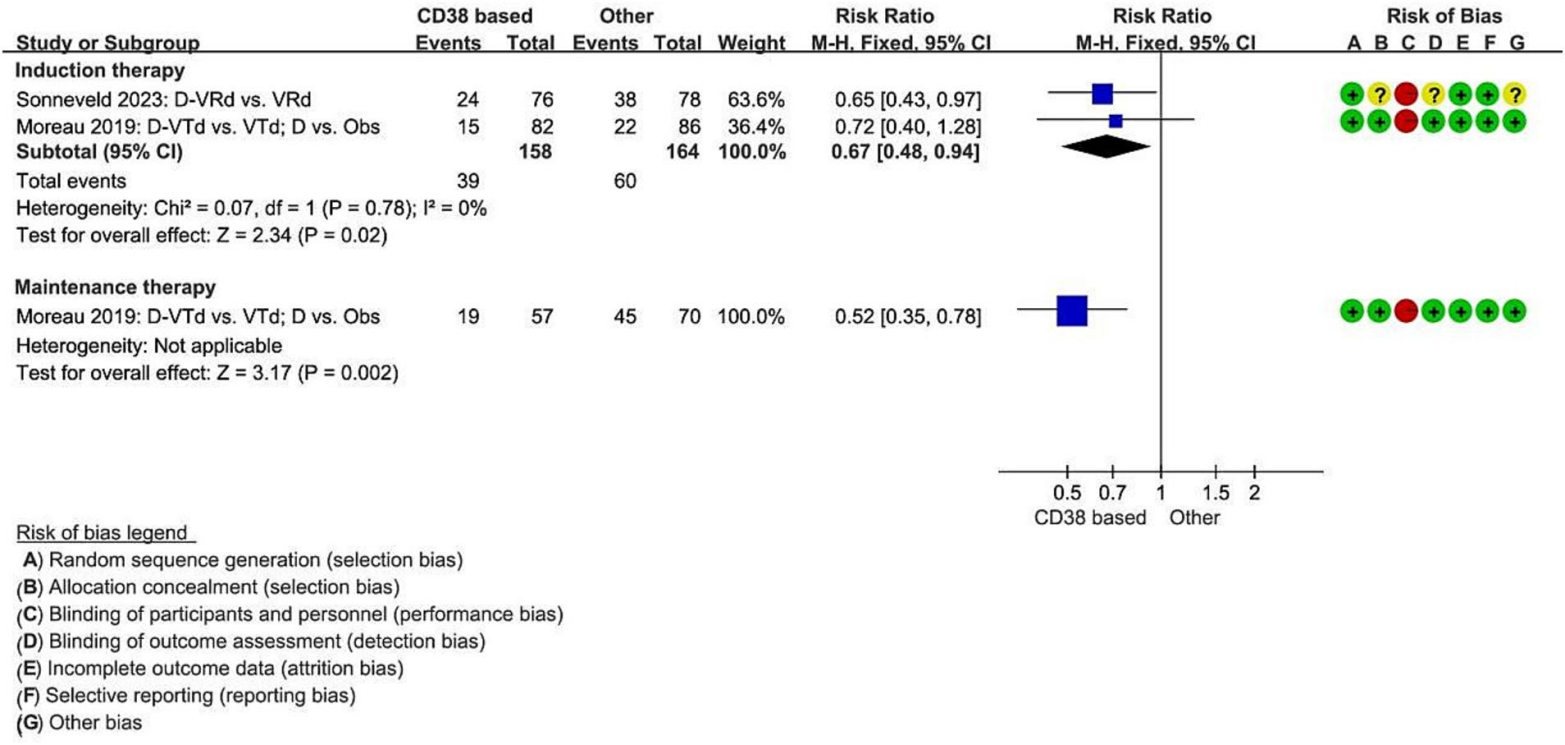
Meta-analysis of disease progression or death associated with CD38-based therapy. D, Daratumumab; VTd, bortezomib, thalidomide, dexamethasone; Obs, Observation only; VRd, lenalidomide, bortezomib, dexamethasone.

Analysis included two studies that reported on disease progression or death during the induction therapy with the regimens D-VTd (Daratumumab, bortezomib, thalidomide, dexamethasone) vs. (versus) VTd (11), and D-RVd (lenalidomide, bortezomib, dexamethasone) vs. RVd (12). Compared to the non-Daratumumab combined group, the Daratumumab combined group significantly reduced the risk of disease progression or death by 33% (RCT = 2, *N* = 322, RR = 0.67, 95%CI 0.48 to 0.94, *p* = 0.02). One study reported the numbers for disease progression or death during the maintenance therapy, for the regimens D vs. Observation only (11). Compared to the Observation only group, the Daratumumab group significantly reduced the risk of disease progression or death by 48% (RCT = 1, *N* = 127, RR = 0.52, 95%CI 0.35 to 0.78, *p* = 0.002, Figure 2).

**Figure 3:**
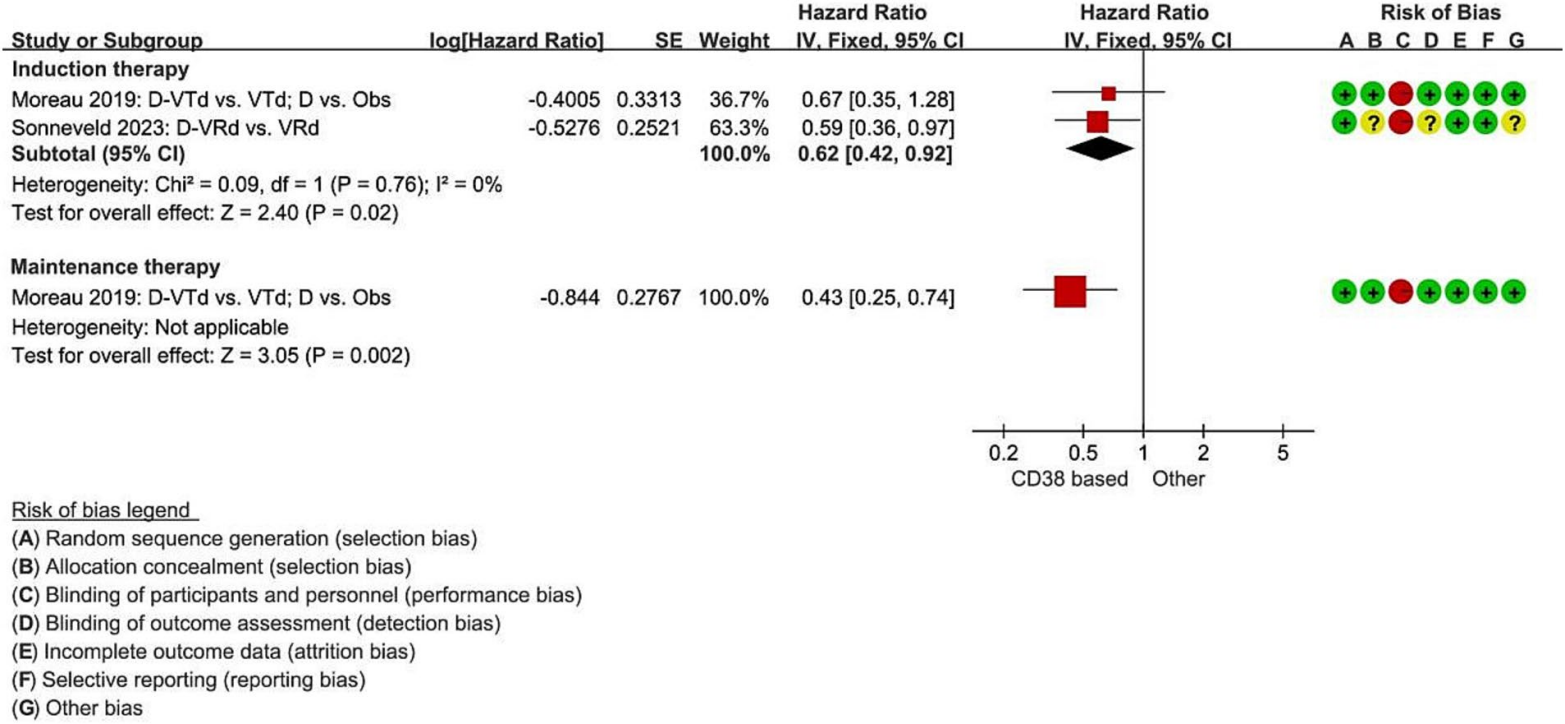
Meta-analysis of hazard ratios for median progression-free survival with CD38-based therapy. D, Daratumumab; VTd, bortezomib, thalidomide, dexamethasone; Obs, Observation only; VRd, lenalidomide, bortezomib, dexamethasone.

Two studies reported the hazard ratio for median PFS during the induction therapy, indicating a significant decrease in the risk of disease progression for the Daratumumab combined group compared to the non-Daratumumab group, with a 38% reduction (RCT = 2, *N* = 322, HR = 0.62, 95%CI 0.42 to 0.92, *p* = 0.02, Figure 3) (11, 12). In the Daratumumab group, the median PFS was not estimable, whereas, in the Observation only group, it was 27.2 months (95%CI 20.7 to 33.6). The use of Daratumumab significantly reduced the risk of disease progression or death by 57% (HR 0.43, 95%CI 0.25 to 0.74, *p* = 0.002, Figure 3).

**Figure 4:**
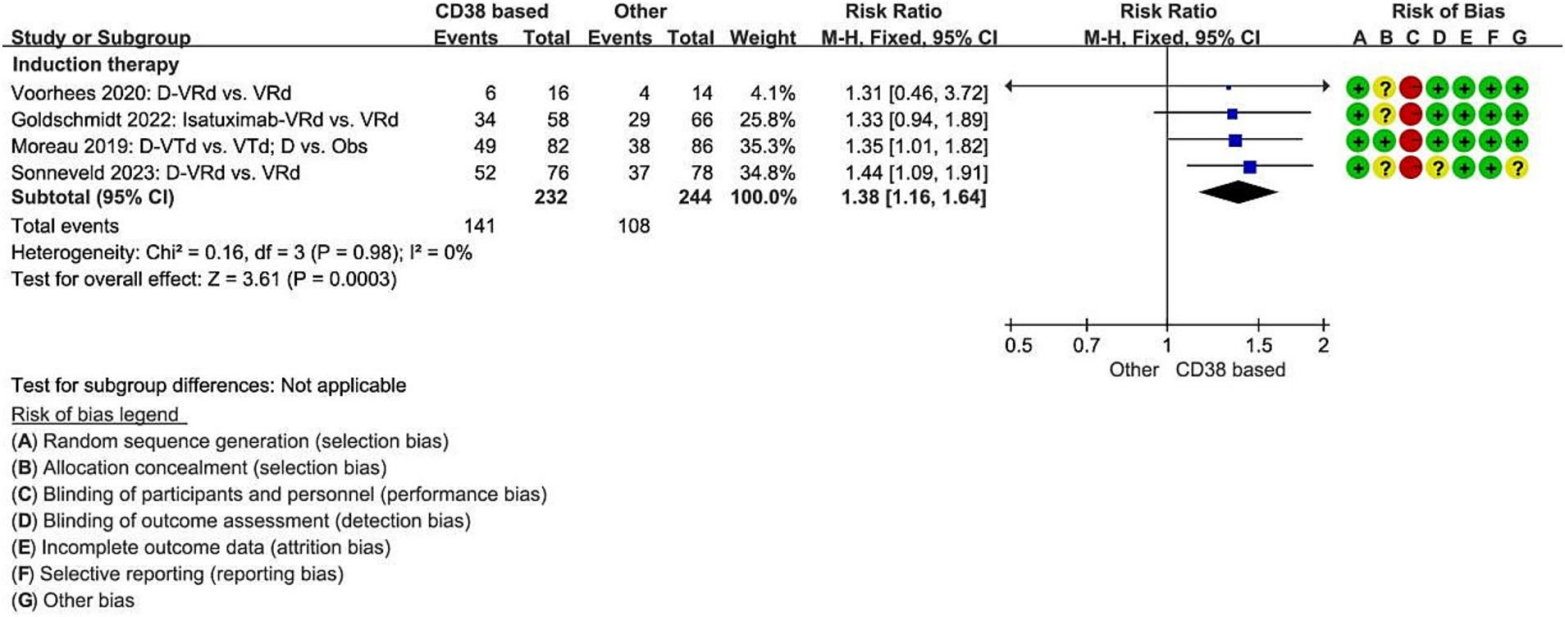
Meta-analysis of the number of patients achieving minimal residual disease negativity with CD38-based therapy. D, Daratumumab; VTd, bortezomib, thalidomide, dexamethasone; Obs, Observation only; VRd, lenalidomide, bortezomib, dexamethasone.

Four studies reported on MRD negativity rates during induction therapy with the regimens Isatuximab-VRd vs. VRd (13), D-VTd vs. VTd (11), and D-VRd vs. VRd (12, 14). The probability of achieving MRD negativity was significantly increased by 38% in the group receiving combination with CD38 compared to the non-CD38 group (RCT = 4, *N* = 476, RR = 1.38, 95%CI 1.16 to 1.64, *p* = 0.0003, Figure 4).

#### 3.2.2 Dual novel drug regimens

A single study comparing the effect of using a dual novel drug regimen during the induction therapy on MRD negativity in high-risk NDMM patients was identified. The treatment regimen compared Isatuximab-KRd (carfilzomib, lenalidomide, dexamethasone) vs. KRd (15). The results suggested that the dual novel drug group could potentially increase the proportion of patients achieving MRD negativity. Utilizing a threshold of 10^−6^, within 2 high-risk cytogenetic abnormalities (HRCA) patients, the use of dual novel drug regimens resulted in a statistically significant 9.05 times higher in MRD negativity compared to the control group (RCT = 1, OR = 9.05, 95% CI 1.57 to 52.14, Supplementary Table S3).

#### 3.2.3 Elotuzumab-based regimens

One study assessed the influence of Elotuzumab during induction therapy on both disease progression or death and overall mortality among high-risk NDMM patients (16). The treatment regimen comparison involved Elotuzumab-RVd vs. RVd. Analysis of the outcomes revealed that there was no statistically significant difference in the risk of disease progression or death between patients treated with Elotuzumab-RVd and those receiving RVd (RCT = 1, *N* = 100, RR = 1.08, 95% CI 0.80 to 1.47, *p* = 0.61, Supplementary Figure S2). At 53 months follow-up, the median PFS for the Elotuzumab-RVd group was 31.47 months (95%CI 18.56 to 53.98), compared to 33.64 months (95%CI 19.55 to not reached) for the RVd group. The overall survival for the Elotuzumab-RVd group was recorded at 68 months (95%CI 61 to 68), while the overall survival for the RVd group was not reached. The difference between the groups was not statistically significant (HR = 0.968, 80% CI 0.697 to 1.344; HR = 1.279, 80% CI 0.819 to 2.000). At 72 months follow-up, the median PFS was 29 months for the Elotuzumab-RVd group and 34 months for the RVd group. The OS for the Elotuzumab-RVd group had not been reached, contrasting with a 68-month OS for the RVd group, also showing no statistically significant difference (HR = 1.11, 80% CI 0.82 to 1.49; HR = 0.85, 80% CI 0.59 to 1.23). No significant difference in the risk of all-cause mortality was observed between the Elotuzumab-RVd and RVd groups during the induction therapy (RCT = 1, *N* = 100, RR = 0.91, 95%CI 0.53 to 1.56, *p* = 0.74, Supplementary Figure S3).

#### 3.2.4 Ixazomib-based regimens

A single study examining the impact of maintenance therapy Ixazomib monotherapy on disease progression or death reported no significant difference between the Ixazomib alone group and the placebo group (17) (RCT = 1, *N* = 115, RR = 0.89, 95%CI 0.68 to 1.15, *p* = 0.36, Supplementary Figure S4). The median PFS during the induction therapy was not reported, and there was no statistically significant difference between the groups (HR = 0.62, 80% CI 0.38 to 1.02).

#### 3.2.5 Carfilzomib-based regimens

Maintenance therapy analysis from two studies comparing KR (carfilzomib, lenalidomide) vs. R (lenalidomide) (18) and KRd vs. R (19) showed no significant difference in the risk of disease progression or death between Carfilzomib combined group and Lenalidomibe alone (RCT = 2, *N* = 148, RR = 0.96, 95%CI 0.63 to 1.47, *p* = 0.86, Supplementary Figure S5). One study also reported that the median PFS during the maintenance therapy in the KRd group was 40.2 months (95%CI 14.7 to 59.1), whereas in the Lenalidomide monotherapy group, it was 34.1 months (95%CI 9.8 to not estimable). There was no statistically significant difference between the groups (HR = 0.74, 95%CI 0.30 to 1.86).

In addition, two studies (19, 20) (KRd vs. R and KCd vs. HSCT) showed no significant difference in the probability of achieving MRD negativity between the Carfilzomib combined group and the Lenalidomide monotherapy or transplant groups (RCT = 2, *N* = 79, RR = 0.78, 95%CI 0.45 to 1.37, *p* = 0.39, Supplementary Figure S6).

### 3.3 For transplant ineligible patients

#### 3.3.1 CD38-based regimens

Three studies assessed the effect of Daratumumab and Isatuximab during the induction therapy on disease progression or death in high-risk NDMM patients, reporting hazard ratios for PFS (21–23). The induction treatment regimens were D-Rd vs. Rd., D-VMP (bortezomib, melphalan, prednisone) vs. VMP and Isatuximab-VRd vs. VRd. The results showed no significant difference in the risk of disease progression or death between CD38 combined group and non-CD38 combined group (RCT = 3, *N* = 264, RR = 1.04, 95%CI 0.78 to 1.39, *p* = 0.77, Supplementary Figure S7).

The median PFS for patients treated with D-VMP was similar to those who were treated with VMP (18 months vs. 18.1 months). Both D-Rd and Isatuximab-VRd groups reached a median PFS that was not reached, but the result of HRs showed no significant difference between the groups (RCT = 3, HR = 0.85, 95%CI 0.59 to 1.24, *p* = 0.41, Supplementary Figure S8).

The isatuximab-based study included a subgroup of patients with HRCA and 1q21 + (defined as having at least three copies of 1q21), however there was no statistically significant difference in either disease progression or death or PFS compared to the control group.

#### 3.3.2 Elotuzumab-based regimens

A single study evaluated the impact of Elotuzumab during the induction therapy on disease progression or death (24). The induction treatment compared Elotuzumab-Rd vs. Rd. Results showed no significant difference in the risk of disease progression or death between the Elotuzumab-Rd and Rd. groups (RCT = 1, *N* = 66, RR = 1.09, 95%CI 0.88 to 1.35, *p* = 0.44, Supplementary Figure S9). The median PFS were not reported in both groups, but the hazard ratio did not reach statistical significance (HR = 0.96, 95%CI 0.56 to 1.64).

#### 3.3.3 Ixazomib-based regimens

Two studies compared the impact of Ixazomib on disease progression or death in high-risk NDMM patients, reporting the hazard ratios for PFS. The first study compared the induction treatment regimen of Ixazomib-Rd vs. Rd (25). The Ixazomib-Rd group demonstrated a significant 16% reduction in the risk of disease progression or death compared to the Rd. group (RCT = 1, *N* = 180, RR = 0.84, 95%CI 0.70 to 1.00, *p* = 0.05). The median PFS was 23.8 months and 18 months in the two groups separately. Compared to the Rd. group, the Ixazomib-Rd group had a 31% lower risk of disease progression or death (HR = 0.690, 95%CI 0.506 to 0.941). The second study compared the maintenance treatment regimens of Ixazomib and Placebo (26). There was no statistically significant difference between the Ixazomib monotherapy group and the placebo group in the risk of disease progression or death (RCT = 1, *N* = 122, RR = 0.92, 95%CI 0.73 to 1.15, *p* = 0.46, Supplementary Figure S10) and median PFS (HR = 1.011, 95%CI 0.631 to 1.621).

#### 3.3.4 Carfilzomib-based regimens

Two studies examined the impact of Carfilzomib used during the induction therapy on disease progression or death with regimens KRd vs. VRd (27) and KMP (carfilzomib, melphalan, prednisone) vs. VMP (28) (bortezomib, melphalan, prednisone).

During the induction therapy, there was no significant difference in the risk of disease progression or death between KRd and VRd groups (RCT = 1, *N* = 255, RR = 0.87, 95%CI 0.63 to 1.20, *p* = 0.39, Supplementary Figure S11). Both studies did not report the median PFS, and there was no statistically significant difference between the groups (RCT = 2, HR = 0.83, 95%CI 0.60 to 1.13, *p* = 0.24, Supplementary Figure S12).

## 4 Discussion

By summarizing the evidence from 18 RCTs (with 1961 patients), this study demonstrates several advantages of the new drug regimens in treating high-risk NDMM patients. CD38-based regimens showed significant improvements in disease progression or death, increased proportion of patients achieving MRD negativity, and improved PFS. Dual novel drug regimens, such as Isatuximab-KRd, also demonstrated a significant increase in MRD negativity. These findings highlight the potential efficacy of these regimens in improving outcomes for high-risk NDMM patients. Notably, we did not identify any studies specifically investigating pomalidomide for NDMM, which may indicate a potential gap in research within this area.

While MRD negativity and PFS improvements are critical markers of therapeutic depth, their prognostic value for OS in high-risk cytogenetic MM requires caution. The IMWG consensus underscores that despite MRD/PFS benefits, aggressive relapse patterns in high-risk disease may attenuate their association with long-term survival. Emerging real-world evidence, such as a retrospective analysis by Li et al. (29), suggests pomalidomide-based regimens (e.g., VPd) may enhance depth of response (≥VGPR: 90.5% vs. 52% with VRd), though comparable 1-year survival rates (OS 90.5% vs. 92%) indicate early response advantages may not immediately translate to survival gains. Prospective RCTs with extended follow-up are needed to validate whether these regimens can durably overcome high-risk cytogenetics.

Achieving MRD negativity is a critical prognostic marker in multiple myeloma, associated with improved survival outcomes irrespective of the treatment regimen or cytogenetic risk status (30). It is widely used and potential surrogate endpoint in multiple myeloma (31). Studies indicate that MRD negativity, especially when sustained, correlates strongly with longer PFS and OS (32). For instance, Isatuximab-based regimens have been shown to achieve higher rates of sustained MRD negativity, which in turn significantly reduces the risk of disease progression or death (33). An important aspect addressed in our analysis is the variability of MRD cut-off values. The variability in MRD cut-off values across different studies poses a challenge in standardizing MRD as a reliable endpoint (34). In our analysis, one study used a threshold of 10^−6^ for MRD cut-off values, which showed significant differences, while using a threshold of 10^−5^ did not yield significant differences (Supplementary Table S3). Despite this, the consensus is that deeper MRD negativity (10^−6^) is more indicative of long-term remission and survival (32).

The findings of this study highlight the need for optimizing treatments for high-risk MM patients. Despite the advancements in drug regimens, there is still room for improvement in terms of achieving better depth of response and prolonging survival outcomes. Individualized treatment approaches should be explored based on the specific genetic and molecular features of high-risk MM patients. The identification of specific cytogenetic abnormalities and genetic mutations can guide treatment selection and help tailor therapy to the underlying disease biology (35). This personalized approach can potentially improve treatment responses and mitigate the adverse effects associated with unnecessary treatments (36).

Combination therapies that target multiple pathways and mechanisms of MM growth and survival should be investigated (37). The results from this meta-analysis suggest that incorporating CD38-targeting agent, into the treatment regimens can significantly improve treatment outcomes in transplant eligible patients. Therefore, further exploration of novel combination therapies, such as the addition of other monoclonal antibodies or targeted agents, may provide additional benefits and overcome treatment resistance. The role of maintenance therapy in HRMM patients should be further evaluated (38). The results from this analysis showed mixed outcomes regarding the efficacy of maintenance therapy in different drug regimens. Further studies are warranted to determine the optimal duration and agents for maintenance therapy in these patients, considering the balance between treatment efficacy and toxicities. Lastly, the development of novel therapeutic strategies, such as immunotherapies and targeted therapies, should be encouraged. Immunotherapies, including chimeric antigen receptor (CAR) T-cell therapy and immune checkpoint inhibitors, have shown promising results in the treatment of MM (39).

The review followed a systematic search and review procedure, ensuring rigorous quality control and minimizing biases. Moreover, this study covers various new drug regimens, including CD38 antibodies, multiple next-generation proteasome inhibitors, immunomodulatory drugs and so on. By comparing the efficacy of different regimes, it can guide clinicians to select the most suitable treatment strategy. Notably, safety profiles (e.g., Grade ≥3 AEs) and response metrics such as objective response rate (ORR) or disease control rate (DCR) were rarely stratified by cytogenetic risk in the included trials. For instance, while studies like CASSIOPEIA and GRIFFIN reported overall ORR, these outcomes were aggregated across risk groups, limiting insights into their applicability for high-risk populations. One of the primary limitations of this systematic review is the focus on short-term outcomes, such as disease progression and death rates, while lacking long-term follow-up data to evaluate the enduring effects of these treatment regimens and patient survival. Furthermore, the number of studies included in the analysis for some regimens is limited, potentially leading to publication bias, and the absence of direct comparisons with non-RCTs restricts the comprehensiveness of our findings. Non-RCTs, such as the GMMG-CONCEPT trial using the Isa-KRd regimen, have provided significant insights, showing comparable efficacy of CD38-targeted therapies in real-world settings (33). However, the heterogeneity in defining high-risk cytogenetic alterations across studies poses a significant limitation. Trials employed varied criteria (Supplementary Table S1), reflecting the evolving understanding of high-risk disease. Modern frameworks, such as the newly updated 2024 mayo Stratification for Myeloma and Risk-Adaptation Tool 4.0 (SMART 4.0) (40) and the International Myeloma Society (IMS) high-risk stratification, incorporate the latest insights into cytogenetic abnormalities, disease stages, and prognostic factors. These differences highlight the dynamic nature of risk classification, challenge the generalizability of findings, and emphasize the need to interpret clinical outcomes within the genomic criteria of each study, tailoring treatment strategies accordingly. Finally, the unique characteristics of high-risk patients, who often exhibit more aggressive disease progression and poorer prognosis, make conducting RCTs challenging. Ethical and practical difficulties arise in enrolling these patients in RCTs, as control groups might receive less effective treatments. Additionally, high-risk patients are more likely to have comorbid conditions and complications that complicate trial design and execution. The limited availability of high-risk patients meeting stringent inclusion criteria further restricts the feasibility of large-scale RCTs. These factors collectively constrain the ability to conduct RCTs, necessitating reliance on observational studies and real-world evidence to supplement clinical trial data.

## 5 Conclusion

This systematic review and meta-analysis highlight the efficacy of new drug regimens in treating newly diagnosed high-risk cytogenetic MM patients. The results indicate significant benefits of CD38-targeted regimens in reducing disease progression or death, increasing the proportion of patients achieving MRD negativity, and improving PFS. Dual novel drug regimens also showed potential in increasing MRD negativity rates. The study reveals differences in outcomes between transplant-eligible and non-transplant-eligible patients, with substantial benefits observed in the former group when using CD38-targeted therapies.

## Data Availability

The original contributions presented in the study are included in the article/Supplementary material, further inquiries can be directed to the corresponding author.
