# Supplementary material for: The efficacy of new drug regimens in treating newly diagnosed high-risk cytogenetic multiple myeloma patients: a systematic literature review and meta-analysis
Source: Front Med (Lausanne). 2025 May 13;12:1575914. doi: 10.3389/fmed.2025.1575914 (PMC12106411; doi:10.3389/fmed.2025.1575914)
Supplement: Supplementary file 1 [file Data_Sheet_1.pdf]

Supplementary Appendix 1. Search strategies, Characteristics of the included study, Results of risk bias assessment

Pubmed

#1. "Multiple Myeloma"[Mesh] OR Multiple Myeloma*[tw] OR Plasma-Cell Myeloma*[tw] OR Myelomatos*[tw] OR Kahler Diseas*[tw] OR Myeloma-Multiple*[tw] OR "morbus kahler"[tw]

#2. "newly diagnos*"[tw] OR untreated[tw] OR "first line"[tw] OR "1ST line"[tw] OR "1 line"[tw] OR "treatment naive"[tw] OR frontline[tw] OR "front line"[tw]

#3. #1 AND #2 5601

#4. "carfilzomib" [Supplementary Concept] OR carfilzomib OR "PR-171" OR Kyprolis OR "fp 101" OR ono 7057 OR "ixazomib" [Supplementary Concept] OR ixazomib OR "mln 2238" OR mln2238 OR Ninlaro OR "pomalidomide" [Supplementary Concept] OR pomalidomide OR Imnovid OR Pomalyst OR actimid OR "cdc 394" OR "daratumumab" [Supplementary Concept] OR daratumumab OR Darzalex OR "humax CD38" OR dalinvi OR "isatuximab" [Supplementary Concept] OR isatuximab OR Sarclisa OR "sar 650984" OR "elotuzumab" [Supplementary Concept] OR elotuzumab OR Empliciti OR huluc63 OR "pdl 063" 4509

#5. #3 AND #4 613

#6. ("controlled clinical trial"[pt] OR "Controlled Clinical Trials as Topic"[MeSH] OR "Random Allocation"[MeSH] OR "Double-Blind Method"[MeSH] OR "single-blind method"[MeSH] OR "Control Groups"[MeSH] OR random*[tiab]) NOT ("Animals"[Mesh] NOT ("Humans"[Mesh] AND "Animals"[Mesh]))

#7. #5 AND #6 122

Embase

#1. 'multiple myeloma'/exp OR ((Multiple NEAR/3 Myeloma*) OR ("Plasma Cell" NEAR/4 (Myeloma* OR tumor OR tumour OR neoplasm*)) OR Myelomatos* OR "Kahler Diseas*" OR "morbus kahler"):ab,ti,kw

#2. ("newly diagnos*" OR untreated OR "first line" OR "1ST line" OR "1 line" OR "treatment naive" OR frontline OR "front line"):ab,ti,kw

#3. #1 and #2 14250

#4. 'carfilzomib'/exp OR 'ixazomib'/exp OR 'pomalidomide'/exp OR 'daratumumab'/exp OR 'isatuximab'/exp OR 'elotuzumab'/exp OR (carfilzomib OR "PR-171" OR Kyprolis OR "fp 101" OR ono 7057 OR ixazomib OR "mln 2238" OR mln2238 OR Ninlaro OR pomalidomide OR Imnovid OR Pomalyst OR actimid OR "cdc 394" OR daratumumab OR Darzalex OR "humax CD38" OR dalinvi OR isatuximab OR Sarclisa OR "sar 650984" OR elotuzumab OR Empliciti OR huluc63 OR "pdl 063"):ab,ti,kw

#5. #3 AND #4 2695

#6. ('controlled clinical trial'/exp OR 'Controlled Clinical Trial (Topic)'/exp OR 'double blind procedure'/de OR 'control group'/de OR 'single blind procedure'/de OR 'triple blind procedure'/de OR 'randomization'/exp OR random*:ab,ti,kw) NOT (('nonhuman'/exp OR 'animal'/exp) NOT 'human'/exp)

#7. #5 AND #6 783

#8. #7 AND 'conference abstract'/it 526

#9. #7 NOT #8 257

Cochrane

#1 MeSH descriptor: [Multiple Myeloma] explode all trees2782

#2 ((Multiple NEAR/3 Myeloma*) OR ("Plasma Cell" NEAR/4 (Myeloma* OR tumor OR tumour OR neoplasm*)) OR Myelomatos* OR "Kahler Diseas*" OR "morbus

kahler"):ti,ab,kw6099

#3 #1 or #26099

#4 ("newly diagnos*" OR untreated OR "first line" OR "1ST line" OR "1 line" OR "treatment naive" OR frontline OR "front line"):ti,ab,kw53251

#5 (carfilzomib OR "PR-171" OR Kyprolis OR "fp 101" OR ono 7057 OR ixazomib OR "mln 2238" OR mln2238 OR Ninlaro OR pomalidomide OR Imnovid OR Pomalyst OR actimid OR "cdc 394" OR daratumumab OR Darzalex OR "humax CD38" OR dalinvi OR isatuximab OR Sarclisa OR "sar 650984" OR elotuzumab OR Empliciti OR huluc63 OR "pdl 063"):ti,ab,kw 1661

#6 #3 and #4 and #5

Study	Random sequence generation (selection bias)	Allocation concealment (selection bias)	Blinding of participants and personnel (performance bias)	Blinding of outcome assessment (detection bias)	Incomplete outcome data (attrition bias)	Selective reporting (reporting bias)	Other bias
Dimopoulos 2019: Ixazomib vs. Placebo	+	+	+	+	+	+	+
Dimopoulos 2020-: Ixazomib vs. Placebo	+	+	+	+	+	+	+
Dimopoulos 2022: Elotuzumab-Rd vs. Rd	+	+	−	+	+	+	−
Dytfeld 2023: KRd vs. R	+	+	−	+	+	+	+
Facon 2019a: D-Rd vs. Rd	+	?	−	+	+	+	+
Facon 2019b: KMP vs. VMP	+	?	−	+	+	+	−
Facon 2021: Ixazomib-Rd vs. Rd	+	?	+	+	+	+	+
Facon 2024: Isatuximab-VRd vs. VRd	+	?	−	+	+	+	+
Francesca 2023: Isatuximab-KRd vs. KRd	+	?	−	+	+	+	+
Gay 2018: KCd vs. KRd	+	?	?	+	+	+	+
Goldschmidt 2022: Isatuximab-VRd vs. VRd	+	?	−	+	+	+	+
Kumar 2020: KRd vs. VRd	+	?	−	+	+	+	+
Mateos 2018: D-VMP vs. VMP	+	+	−	+	+	+	+
Mina 2023: KRd+ASCT vs. KCd+ASCT; KR vs. R	+	+	−	+	+	+	+
Moreau 2019: D-VTd vs. VTd; D vs. Obs	+	+	−	+	+	+	+
Sonneveld 2023: D-VRd vs. VRd	+	?	−	?	+	+	?
Usmani 2021: Elotuzumab-RVd vs. RVd	+	+	−	+	+	+	+
Voorhees 2020: D-VRd vs. VRd	+	?	−	+	+	+	+
Yong 2023: KCd vs. HSCT	+	+	−	+	+	+	+

Supplementary Appendix 2. Results of different treatment regimens

For Transplant Eligible Patients

Table S3. Results of minimal residual disease negativity in different patients

Study ID	Treatment	Population	Method	n/N; OR (95%CI)	
Francesca 2023	Isa-KRd vs. KRd	1 HRCA	10^{-5}: NGS.	I: 38/49; C: 32/49	1.86 (0.76-4.57)
Francesca 2023	Isa-KRd vs. KRd	2 HRCA	10^{-5}: NGS.	I: 10/13; C: 8/15	2.76 (0.52-14.56)
Francesca 2023	Isa-KRd vs. KRd	1 HRCA	10^{-6}: NGS.	I: 34/49; C: 26/49	2.04 (0.88-4.70)
Francesca 2023	Isa-KRd vs. KRd	2 HRCA	10^{-6}: NGS.	I: 10/13; C: 4/15	9.05 (1.57-52.14)

CI, confidence interval; Isa, isatuximab; HRCA, high-risk cytogenetic abnormalities; K, carfilzomib; R, lenalidomide; d, dexamethasone; NGS, next-generation sequencing; OR, odds ratio; MRD, minimal residual disease.

Figure S2. Results of progression or death with Elotuzumab-based regimens

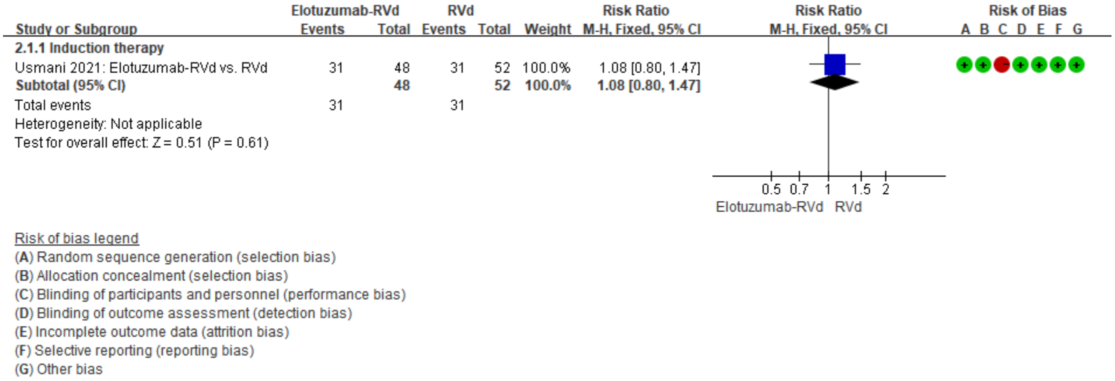

RVd, lenalidomide, bortezomib, dexamethasone;

Figure S3. Results of all-cause mortality with Elotuzumab-based regimens

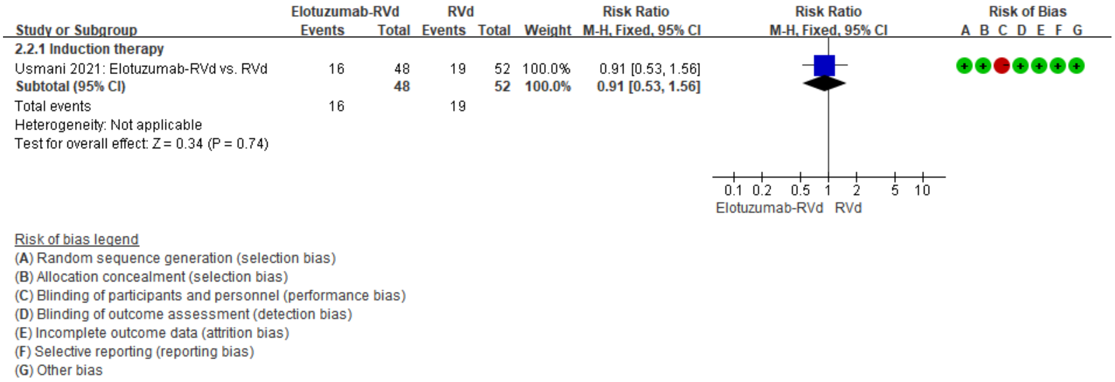

RVd, lenalidomide, bortezomib, dexamethasone;

Figure S4. Results of progression or death with Ixazomib-based regimens

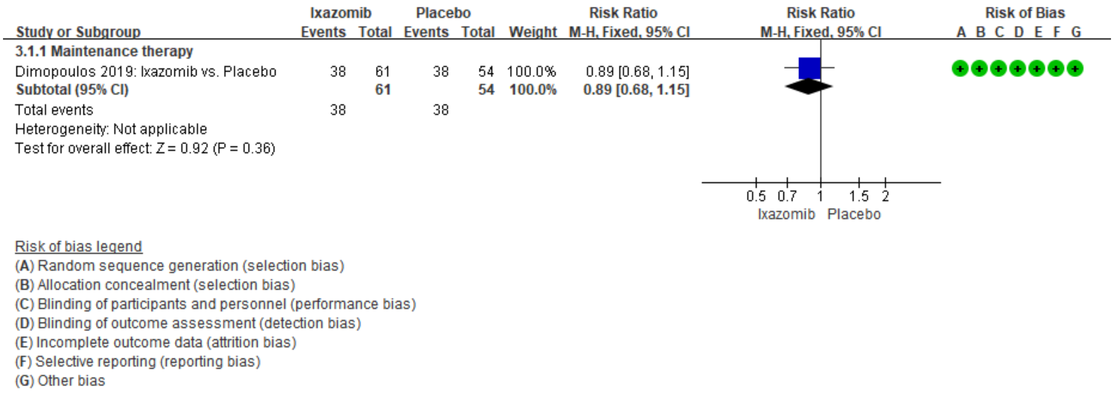

Figure S5. Results of progression or death with Carfilzomib-based regimens

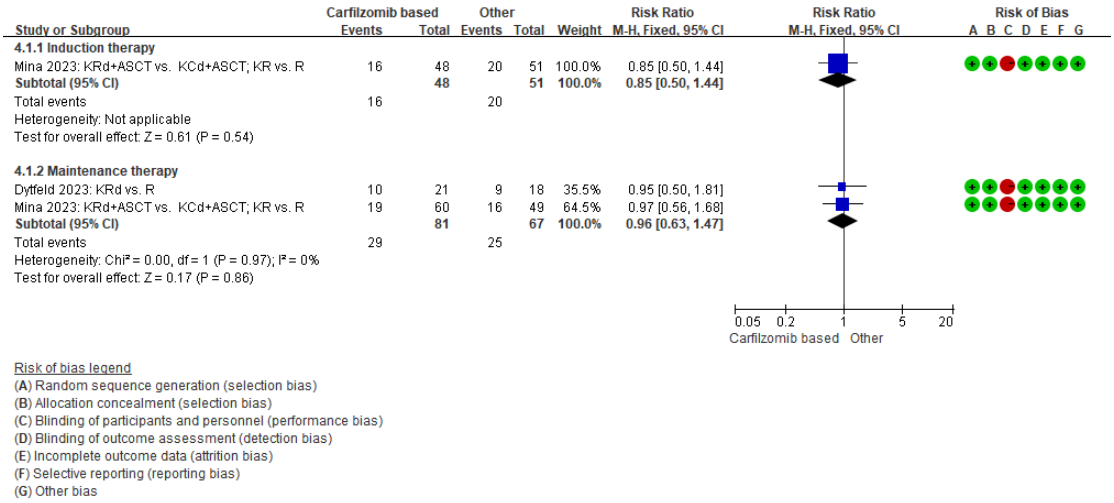

Figure S6. Results of the number of minimal residual disease negative patients with Carfilzomib-based regimens

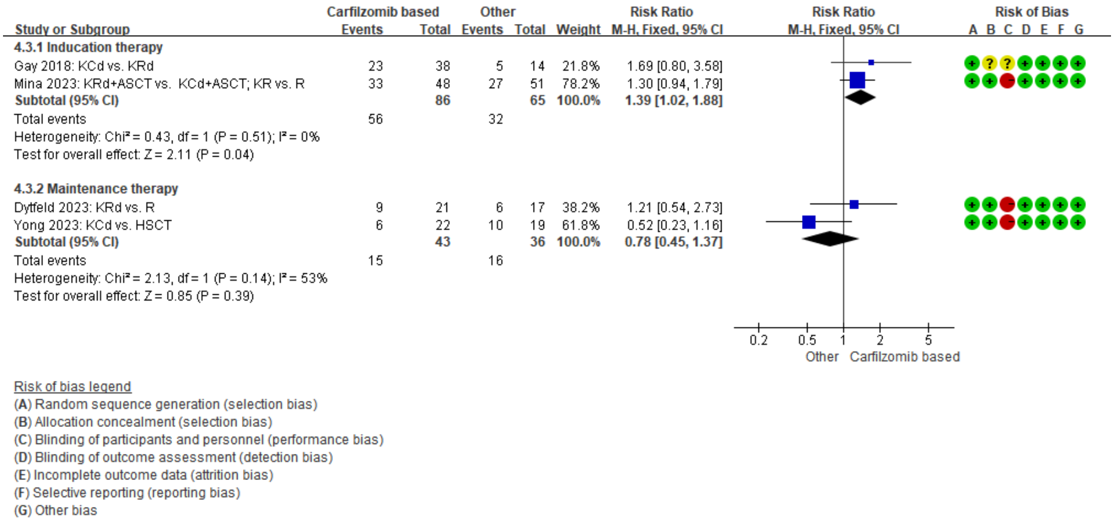

Figure S7. Results of all-cause mortality with Carfilzomib-based regimens

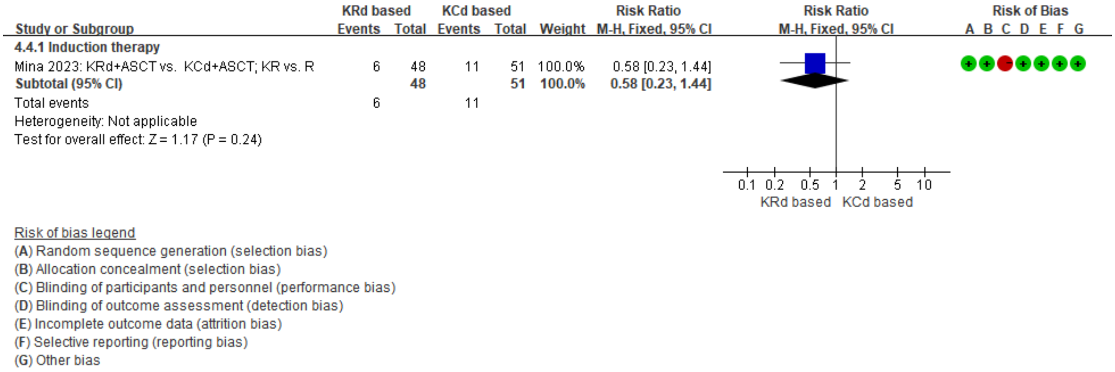

KRd, carfilzomib, lenalidomide, dexamethasone; KCd, carfilzomib, cyclophosphamide, dexamethasone

For Non-transplant Eligible Patients

Figure S8. Results of progression or death with CD38-based regimens

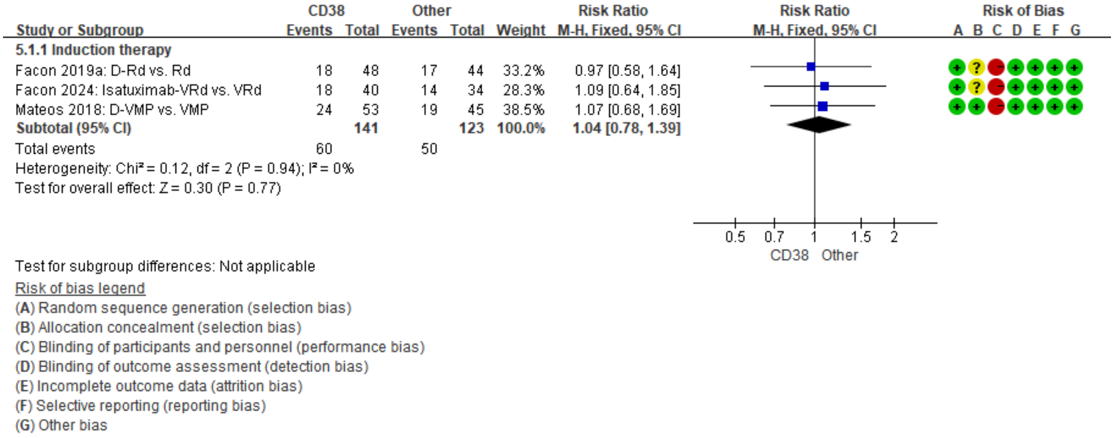

Figure S9. Results of median progression-free survival on hazard ratios with CD38-based regimens

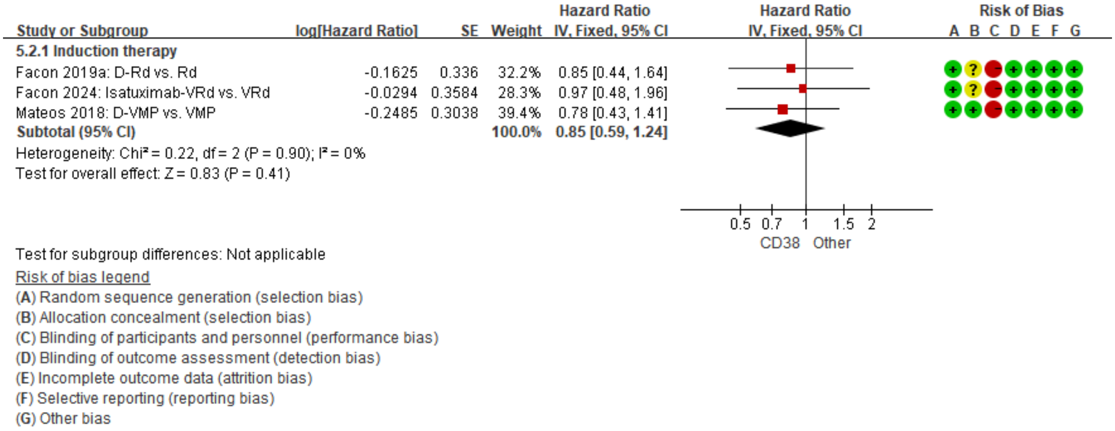

Figure S10. Results of progression or death with Elotuzumab-based regimens

Study or Subgroup	Elotuzumab-Rd Events	Elotuzumab-Rd Total	Rd Events	Rd Total	Weight	Risk Ratio M-H, Fixed, 95% CI	Risk of Bias A B C D E F G
**6.1.1 Induction therapy**							
Dimopoulos 2022: Elotuzumab-Rd vs. Rd	27	31	28	35	100.0%	1.09 [0.88, 1.35]	+ + - + + + -
**Subtotal (95% CI)**		**31**		**35**	**100.0%**	**1.09 [0.88, 1.35]**	
Total events	27		28				
Heterogeneity: Not applicable							
Test for overall effect: Z = 0.78 (P = 0.44)							

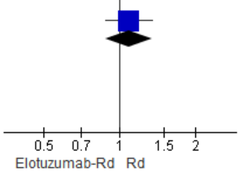

Risk of bias legend
(A) Random sequence generation (selection bias)
(B) Allocation concealment (selection bias)
(C) Blinding of participants and personnel (performance bias)
(D) Blinding of outcome assessment (detection bias)
(E) Incomplete outcome data (attrition bias)
(F) Selective reporting (reporting bias)
(G) Other bias

Rd, lenalidomide, dexamethasone

Figure S11. Results of progression or death with Ixazomib-based regimens

Study or Subgroup	Ixazomib based Events	Ixazomib based Total	Other Events	Other Total	Weight	Risk Ratio M-H, Fixed, 95% CI	Risk of Bias A B C D E F G
**7.1.1 Induction therapy**							
Facon 2021: Ixazomib-Rd vs. Rd	80	134	104	146	100.0%	0.84 [0.70, 1.00]	+ ? + + + + +
**Subtotal (95% CI)**		**134**		**146**	**100.0%**	**0.84 [0.70, 1.00]**	
Total events	80		104				
Heterogeneity: Not applicable							
Test for overall effect: Z = 2.00 (P = 0.05)							
**7.1.2 Maintenance therapy**							
Dimopoulos 2020-: Ixazomib vs. Placebo	51	74	36	48	100.0%	0.92 [0.73, 1.15]	+ + + + + + +
**Subtotal (95% CI)**		**74**		**48**	**100.0%**	**0.92 [0.73, 1.15]**	
Total events	51		36				
Heterogeneity: Not applicable							
Test for overall effect: Z = 0.74 (P = 0.46)							

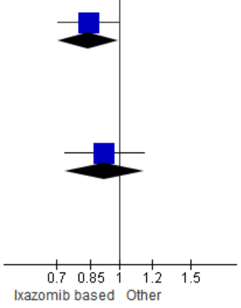

Risk of bias legend
(A) Random sequence generation (selection bias)
(B) Allocation concealment (selection bias)
(C) Blinding of participants and personnel (performance bias)
(D) Blinding of outcome assessment (detection bias)
(E) Incomplete outcome data (attrition bias)
(F) Selective reporting (reporting bias)
(G) Other bias

Figure S12. Results of progression or death with Carfilzomib-based regimens

Study or Subgroup	Carfilzomib based Events	Carfilzomib based Total	Other Events	Other Total	Weight	Risk Ratio M-H, Fixed, 95% CI	Risk of Bias A B C D E F G
**8.1.1 Induction therapy**							
Kumar 2020: KRd vs. VRd	43	127	50	128	100.0%	0.87 [0.63, 1.20]	+ ? - + + + +
**Subtotal (95% CI)**		**127**		**128**	**100.0%**	**0.87 [0.63, 1.20]**	
Total events	43		50				
Heterogeneity: Not applicable							
Test for overall effect: Z = 0.86 (P = 0.39)							

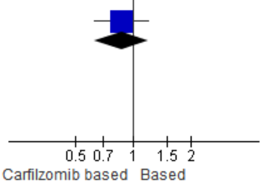

Risk of bias legend
(A) Random sequence generation (selection bias)
(B) Allocation concealment (selection bias)
(C) Blinding of participants and personnel (performance bias)
(D) Blinding of outcome assessment (detection bias)
(E) Incomplete outcome data (attrition bias)
(F) Selective reporting (reporting bias)
(G) Other bias

Figure S13. Results of median progression-free survival on hazard ratios with Carfilzomib-based regimens

Study or Subgroup	log[Hazard Ratio]	SE	Weight	Hazard Ratio IV, Fixed, 95% CI	Risk of Bias A	B	C	D	E	F	G
**8.2.1 Induction therapy**											
Facon 2019b: KMP vs. VMP	-0.0408	0.2571	39.3%	0.96 [0.58, 1.59]	+	?	-	+	+	+	-
Kumar 2020: KRd vs. VRd	-0.2877	0.2069	60.7%	0.75 [0.50, 1.13]	+	?	-	+	+	+	+
**Subtotal (95% CI)**			**100.0%**	**0.83 [0.60, 1.13]**							
Heterogeneity: Chi² = 0.56, df = 1 (P = 0.45); I² = 0%											
Test for overall effect: Z = 1.18 (P = 0.24)											

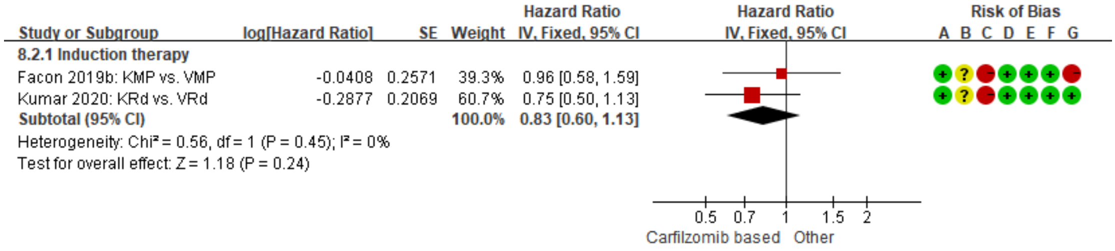

Risk of bias legend
(**A**) Random sequence generation (selection bias)
(**B**) Allocation concealment (selection bias)
(**C**) Blinding of participants and personnel (performance bias)
(**D**) Blinding of outcome assessment (detection bias)
(**E**) Incomplete outcome data (attrition bias)
(**F**) Selective reporting (reporting bias)
(**G**) Other bias
