# Supplementary material for: The efficacy of new drug regimens in treating newly diagnosed high-risk cytogenetic multiple myeloma patients: a systematic literature review and meta-analysis
Source: Front Med (Lausanne). 2025 May 13;12:1575914. doi: 10.3389/fmed.2025.1575914 (PMC12106411; doi:10.3389/fmed.2025.1575914)
Supplement: Supplementary file 2 [file Table_1.docx]

| **Table S1 Characteristics of the included study** | | | | |
| --- | --- | --- | --- | --- |
| **Study ID** | **Phase of trial** | **Trial registration number;  Trial Name** | **Country** | **Definition of high risk** |
| Dytfeld 2023 | 3 | NCT02659293 | USA and Poland | Cytogenetic risk factors (presence vs absence of del(13) (q14), t(4;14)(p16;q32), t(14;16)(q32;q23), del(17)(p13.1), or hypodiploidy), and site location (USA vs Poland). |
| Dimopoulos 2022 | 3 | NCT01335399; ELOQUENT-1 | USA, Australia, Austria, Belgium, Canada, Czechia, Germany, Greece, Hungary, Ireland, Israel, Italy, Poland, Puerto Rico, Romania, Russian Federation, Spain, Switzerland, Turkey, UK | High-risk disease was defined as International Staging System stage II or III and t(4;14) or del(17p) mutations. |
| Dimopoulos 2020 | 3 | NCT02312258; TOURMALINE-MM4 | USA, Argentina, Australia, Austria, Belgium, Brazil, Canada, Chile, China, Colombia, Croatia, Czechia, Denmark, France, Germany, Greece, Hungary, Israel, Italy, Japan, Korea, Republic of, Mexico, Poland, Portugal, Russian Federation, Serbia, Singapore, South Africa, Spain, Sweden, Switzerland, Taiwan, Thailand, Turkey, UK | High-risk cytogenetic abnormalities were del(17p), t(4;14), and t(14;16). If all three abnormalities were unknown, indeterminate, or missing, the patient was called unclassifiable. There was no cutoff for defining the presence of del(17p). |
| Dimopoulos 2019 | 3 | NCT02181413; TOURMALINE-MM3 | Europe, the Middle East, Africa, Asia, and North and South America | High-risk cytogenetic abnormalities were detected by fluorescence in-situ hybridisation or karyotype analysis and were defined as del(17p), t(4;14), and t(14;16). If all three abnormalities were unknown, indeterminate or missing, the patient was called unclassifiable. There was no cutoff for defining the presence of del(17p). |
| Facon 2024 | 3 | NCT03319667 | Australia, Belgium, China, Czech Republic, Germany, Denmark, France, Greece, Italy, Japan, Lithuania, Mexico, Mew Zealand, Poland, Portugal, Russian Federation, Spain, Sweden, Turkey, USA | High cytogenetic risk was defined as the presence of del(17p), t(4;14), t(14;16), or a combination of these, with cutoffs defined below. |
|  |  |  |  | HRCA defined as del(17p), and/or t(4;14), and/or t(14;16). 1q21+ abnormality defined as at least 3. |
| Facon 2021 | 3 | NCT01850524; TOURMALINE-MM2 | Europe, North America, and Asia-Pacific | Patients with expanded high-risk cytogenetic abnormalities defined as del(17p), t(4;14), t(14;16), and amp(1q21), which was selected based on data indicating the particular benefit of PI-based treatment in this subgroup. |
| Facon 2019a | 3 | NCT02252172; MAIA | North America, Europe, the Middle East, and the Asia-Pacific region | Cytogenetic risk was based on fluorescence in situ hybridization or karyotype analysis; patients who had a high-risk cytogenetic profile had at least one high-risk abnormality (del17p, t[14;16], or t[4;14]). |
| Facon 2019b | 3 | NCT01818752; CLARION | North America, Europe, the Asia-Pacific, and other regions (Mexico, Argentina, Israel) | The high-risk group consisted of patients with the genetic subtypes t(4;14), t(14;16), or deletion 17p in ≥60% of plasma cells. |
| Goldschmidt 2022 | 3 | NCT03617731; GMMG-HD7 | Germany | High-risk cytogenetics defined as the presence of at least one of the following mutations: del(17)(p13), t(4;14)(p16;q32), or t(14;16)(q32;q23). |
| Gay 2018 | NR | NR | NR | With high-risk chromosomal abnormalities [del17 and/or t(4;14) and/or t(14:16)detected by FISH] |
| Kumar 2020 | 3 | NCT01863550; ENDURANCE; E1A11 | USA | Metaphase cytogenetics was considered positive in the presence of any abnormality. |
| Mina 2023 | 2 | NCT02203643; UNITO-MM-01/FORTE | Italy | 1 HRCA was defined as the presence of one of the following high-risk cytogenetic abnormalities: del(17p), t(4;14), t(14;16), del(1p), gain(1q), or amp(1q); |
| Moreau 2019 | 3 | NCT02541383; CASSIOPEIA | European sites | These patients had at least one high-risk abnormality: del17p (≥50% abnormal cells) or t(4;14) (≥30% abnormal cells). |
| (Moreau 2021) |  |  |  |  |
| Mateos 2018 | 3 | NCT02195479; ALCYONE | North and South America, Europe, and the Asia - Pacific region | A high-risk cytogenetic profile was defined by a finding of t(4;14), t(14;16), or del17p on fluorescence in situ hybridization testing or a finding of t(4;14) or del17p on karyotype testing. |
| Usmani 2021 | 2 | NCT01668719; SWOG-1211 | USA | Patients had to have high-risk multiple myeloma on the basis of one or more of the following criteria at the time of initial diagnosis (before any chemotherapy): (1) poor risk genomic signature according to the University of Arkansas 70-gene model (available clinically as MyPRS score; Signal Genetics [San Diego, CA, USA]; here, referred to as GEPhi); (2) translocations t(14;16) or t(14;20) or deletion del(17p) by FISH or cytogenetics, or gain (three copies) or amplification (more than three copies) of chromosome 1q21 by FISH (standard percentage cutoff values for each type of FISH test abnormality were used in local laboratories; typically 5%, but ranging from 1.5% to 7.5%); (3) primary plasma cell leukaemia (defined by either ≥2000 plasma cells per mL of peripheral blood, or 20% on a manual differential count);12 and (4) serum lactate dehydrogenase two times or more the institutional upper limit of normal. |
| (Usmani 2022) |  |  |  |  |
| Voorhees 2020 | 2 | NCT02874742; the GRIFFIN trial | USA | Cytogenetic risk was assessed by FISH (local testing); high risk was defined as the presence of del17p, t(4;14), or t(14;16) among patients with available cytogenetic risk data. |
| Yong 2023 | 2 | NCT02315716; CARDAMON | UK | High risk is defined as any ONE of the following findings on FISH analysis of selected CD138+ cells: 1. IgH translocation t(4;14), t(14;16) or t(14;20); 2. Deletion 17p (if present in ≥50% of cells);3. 1p deletion and /or 1q gain. |
| NR, not reported; FISH, fluorescence in situ hybridization; HRCA, high-risk cytogenetic abnormalities | | | | |
