# Supplementary material for: The efficacy of new drug regimens in treating newly diagnosed high-risk cytogenetic multiple myeloma patients: a systematic literature review and meta-analysis
Source: Front Med (Lausanne). 2025 May 13;12:1575914. doi: 10.3389/fmed.2025.1575914 (PMC12106411; doi:10.3389/fmed.2025.1575914)
Supplement: Supplementary file 3 [file Table_2.docx]

| **Table S2. Treatment regimens of the included studies** | | | | | |
| --- | --- | --- | --- | --- | --- |
| **Study ID** | **Sample size** | **Transplant-Eligible/Ineligible** | **Treatment phase** | **Protocol abbreviation** | **Treatment protocol** |
| Dytfeld 2023 | 39 | Transplant-Eligible NDMM | Maintenance therapy | KRd vs. R | Patients in the carfilzomib, lenalidomide, and dexamethasone group received 8-36 cycles of carfilzomib 20 mg/m^2^ administered intravenously in cycle one on days 1 and 2 then 36 mg/m^2^ on days 1, 2, 8, 9, 15, and 16 in cycles one to four and 36 mg/m^2^ on days 1, 2, 15, and 16 from cycle five up to 36 cycles; lenalidomide 25 mg administered orally on days 1–21; and dexamethasone 20 mg administered orally on days 1, 8, 15, and 22 in 28-day cycles. To limit the risk of toxic effects and financial burden of the treatment, patients in the carfilzomib, lenalidomide, and dexamethasone group with no detectable minimal residual disease after cycle six (defined by the IMWG as at least 10-5 sensitivity and at least a complete response) and protocol-defined, standard-risk cytogenetics (ie, no high-risk cytogenetic features) were switched to lenalidomide maintenance (best tolerated dose up to 15 mg) as of cycle nine, as part of the risk-adapted and minimal residual disease-directed study design. |
| Dimopoulos 2022 | 66 | Transplant-Ineligible NDMM | Induction therapy | Elotuzumab-Rd vs. Rd | Patients in the elotuzumab plus lenalidomide and dexamethasone group received elotuzumab administered intravenously at a dose of 10 mg/kg on days 1, 8, 15, and 22 during cycles 1 and 2, days 1 and 15 during cycles 3-18, and then at a dose of 20 mg/kg on day 1 for subsequent cycles. In both treatment groups, patients received 25 mg lenalidomide orally on days 1–21 of each cycle and 40 mg dexamethasone on days 1, 8, 15, and 22 of each cycle (on the weeks of elotuzumab administration, patients in the elotuzumab plus lenalidomide and dexamethasone group were given dexamethasone 28 mg orally and 8 mg intravenously). |
| Dimopoulos 2020 | 122 | Transplant-Ineligible NDMM | Maintenance therapy | Ixazomib vs. Placebo | Patients were randomly assigned 3:2 to receive oral ixazomib 3 mg or placebo on days 1, 8, and 15 of 28-day cycles. The dose was increased to 4 mg from cycle 5 if tolerated during cycles 1-4. |
| Dimopoulos 2019 | 115 | Transplant-Eligible NDMM | Maintenance therapy | Ixazomib vs. Placebo | Patients received oral ixazomib or placebo 3 mg on days 1, 8, and 15 in 28 ­day cycles. Dose was increased to 4 mg from cycle 5 if tolerated during cycles 1-4. |
| Facon 2024 | 74 | Transplant-Ineligible NDMM | Induction therapy | Isatuximab-VRd vs. VRd | Four induction cycles (with 6 weeks per cycle) were followed by 4-week cycles of continuous |
|  |  |  |  |  | treatment with isatuximab-Rd (in the isatuximab VRd group) or Rd (in the VRd group) (see below), until the occurrence of disease progression, an unacceptable adverse event, or other discontinuation criteria. |
|  |  |  |  |  | During induction, all patients received VRd, which consisted of subcutaneous bortezomib (1.3 mg per square meter on days 1, 4, 8, 11, 22, 25, 29, and 32), oral lenalidomide (25 mg per day |
|  |  |  |  |  | [or 10 mg per day if the estimated GFR was 30 to <60 ml per minute per 1.73 m2 ] on days 1 to |
|  |  |  |  |  | 14 and 22 to 35), and oral or intravenous dexamethasone (20 mg per day on days 1, 2, 4, 5, 8, |
|  |  |  |  |  | 9, 11, 12, 15, 22, 23, 25, 26, 29, 30, 32, and 33 [or on days 1, 4, 8, 11, 15, 22, 25, 29, and 32 in |
|  |  |  |  |  | patients ≥75 years of age]). Patients in the isatuximab-VRd group received intravenous isatuximab (10 mg per kilogram of body weight once weekly in cycle 1, with subsequent cycles occurring every 2 weeks). Antibacterial prophylactic treatment was recommended for all patients during induction. |
| Facon 2021 | 280 | Transplant-Ineligible NDMM | Induction therapy | Ixazomib-Rd vs. Placebo-Rd | Oral ixazomib 4 mg or placebo on days 1, 8, and 15, plus oral lenalidomide 25 mg on days 1 to 21 (10 mg for patients with creatinine clearance [CrCl] ≤60 or ≤50 mL/min, depending on local prescribing information) and oral dexamethasone 40 mg on days 1, 8, 15, and 22 (reduced to 20 mg in patients aged .75 years at randomization) of each dosing cycle in 28-day cycles. |
|  |  |  |  |  | Patients continued treatment for 18 cycles or until progressive disease (PD) or unacceptable toxicity, whichever came first. After 18 cycles, dexamethasone was discontinued, and patients continued the assigned drug regimen with reduced dose levels of ixazomib 3 mg and lenalidomide 10 mg until progression or unacceptable toxicity. |
| Facon 2019a | 92 | Transplant-Ineligible NDMM | Induction therapy | Daratumumab-Rd vs. Rd | During each 28-day cycle, all the patients received oral lenalidomide (25 mg on days 1 through 21) and oral dexamethasone (40 mg on days 1, 8, 15, and 22) until disease progression or unacceptable toxic effects. For patients who had a creatinine clearance between 30 and 50 ml per minute, a reduced dose of lenalidomide (10 mg) was recommended. Adjustment of the dose of lenalidomide was recommended in the case of neutropenia and thrombocytopenia. Patients who were older than 75 years of age or who had a body-mass index (the weight in kilograms divided by the square of the height in meters) of less than 18.5 received dexamethasone at a dose of 20 mg once weekly. Patients in the daratumumab group received intravenous daratumumab at a dose of 16 mg per kilogram of body weight once weekly during cycles 1 and 2, every 2 weeks during cycles 3 through 6, and every 4 weeks thereafter; preinfusion medications were administered approximately 1 hour before each daratumumab dose (details are provided in the Supplementary Appendix). |
| Facon 2019b | 121 | Transplant-Ineligible NDMM | Induction therapy | KMP vs. VMP | **KMP:** Patients received carfilzomib administered as a 30-minute IV infusion on days 1, 2, 8, 9, 22, 23, 29, and 30 (20 mg/m2 on days 1 and 2 of cycle 1; 36 mg/m2 thereafter) on days 1, 4, 8, 11, 22, 25, 29, and 32 (days 4, 11, 25, and 32 were omitted for cycles 5-9). IV hydration (250-500 mL) was administered before and after carfilzomib dosing during cycle 1 and at the investigator's discretion for cycles ≥ 2. Dexamethasone (4 mg, IV or oral) was administered on days 8, 9, 22, 23, 29, and 30 before carfilzomib dosing during cycle 1 only. Patients received melphalan (9 mg/m2; oral) and prednisone (60 mg/m2; oral) on days 1-4 in each cycle. Antiviral prophylaxis was administered daily to all patients for the duration of treatment. |
|  |  |  |  |  | VMP: Patients received bortezomib (1.3 mg/m2; 3- to 5-second IV bolus or subcutaneous injection per investigator’s choice, dose modification, or regulatory approval) on days 1, 4, 8, 11, 22, 25, 29, and 32 (days 4, 11, 25, and 32 were omitted for cycles 5-9). IV hydration (250-500 mL) was administered before and after carfilzomib dosing during cycle 1 and at the investigator's discretion for cycles ≥ 2. Dexamethasone (4 mg, IV or oral) was administered on days 8, 9, 22, 23, 29, and 30 before carfilzomib dosing during cycle 1 only. Patients received melphalan (9 mg/m2; oral) and prednisone (60 mg/m2; oral) on days 1-4 in each cycle. Antiviral prophylaxis was administered daily to all patients for the duration of treatment. |
| Goldschmidt 2022 | 124 | Transplant-Eligible NDMM | Induction therapy | Isatuximab-VRd vs. VRd | Isatuximab group also received isatuximab (10 mg/kg intravenously on days 1, 8, 15, 22, and 29 of cycle 1 and on days 1, 15, and 29 of cycles 2 and 3). Lenalidomide (25 mg orally on days 1-14 and 22-35), bortezomib (1.3 mg/m² subcutaneously on days 1, 4, 8, 11, 22, 25, 29, and 32), and dexamethasone (20 mg orally on days 1-2, 4-5, 8-9, 11-12, 15, 22-23, 25-26, 29-30, and 32-33). |
| Gay 2018 | 154 | Transplant-Eligible NDMM | Induction therapy | KCd vs. KRd | **KCd:** 4 28-day induction cycles with KCd (carfilzomib20/36 mg/m^2^ IV days 1,2,8,9,15,16; cyclophosphamide 300 mg/m2 days1,8,15; dexamethasone 20 mg days 1,2,8,9,15,16) followed by MEL200ASCT and consolidation with 4 KCd cycles. |
|  |  |  |  |  | KRd: 4 28-day cycles withKRd (carfilzomib 20/36 mg/m2 IV days 1,2,8,9,15,16; lenalidomide 25 mgdays 1-21; dexamethasone 20 mg days 1,2,8,9,15,16) followed by MEL200-ASCT and 4 KRd cycles. |
| Kumar 2020 | 255 | Transplant-Ineligible NDMM | Induction therapy | KRd vs. VRd | **KRd:** For nine cycles of 4 weeks, patients in the KRd group received 36 mg/m² of intravenous carfilzomib on days 1, 2, 8, 9, 15, and 16 (carfilzomib was given at 20 mg/m² on day 1 and day 2 of cycle one), along with lenalidomide 25 mg orally once daily on days 1–21 of all cycles, and dexamethasone 40 mg orally once weekly on days 1, 8, 15, and 22 of cycles one to four. The dose of dexamethasone was reduced to 20 mg on days 1, 8, 15, and 22 of cycle five to cycle nine. These procedures translate to 27 weeks of lenalidomide treat ment in the KRd group during 36 weeks of induction therapy. |
|  |  |  |  |  | VRd: For 12 cycles of 3 weeks, patients in the VRd group received bortezomib 1·3 mg/m² administered subcutaneously or intravenously on days 1, 4, 8, and 11 of cycles 1–8 and day 1 and day 8 of cycle nine to cycle 12, lenalidomide 25 mg orally on days 1–14, and dexamethasone 20 mg orally on days 1, 2, 4, 5, 8, 9, 11, and 12.The dose of dexamethasone was reduced to 10 mg on days 1, 2, 4, 5, 8, 9, 11, and 12 of cycle five to cycle eight and on days 1, 2, 8, and 9 of cycle nine to cycle 12. These procedures translate to 24 weeks of lenalidomide treatment in the VRd group during 36 weeks of induction therapy. |
| Mina 2023 | 138 | Transplant-Eligible NDMM | Induction therapy | KRd + ASCT vs. KRd12 vs. KCd+ASCT | **KRd + ASCT:** Patients in the KRd plus ASCT group received four 28-day induction cycles with KRd, ASCT conditioned with melphalan at 200 mg/m² (MEL200-ASCT), and four KRd consolidation cycles. |
|  |  |  |  |  | KRd 12: Patients in the KRd12 group received four 28-day induction cycles with KRd, stem-cell mobilisation and collection, and eight KRd consolidation cycles without upfront MEL200-ASCT. |
|  |  |  |  |  | KCd + ASCT: Patients in the KCd plus ASCT group received four 28-day induction cycles with KCd, MEL200-ASCT, and four KCd consolidation cycles. |
|  | 109 | Transplant-Eligible NDMM | Maintenance therapy | KR vs. R | With or withoput carfilzomib 36 mg/m² was administered intravenously on days 1-2 and days 15-16, every 28 days for up to 2 years, and lenalidomide 10 mg was administered orally on days 1-21 every 28 days until progression or intolerance in both group. |
| Moreau 2019 | 168 | Transplant-Eligible NDMM | Induction therapy | Daratumumab-VTd vs. VTd | All patients received up to four 28-day, pre-transplant induction cycles and two 28-day, post-transplant consolidation cycles of subcutaneous bortezomib (1.3 mg/m² twice per week in week 1 [days 1 and 4] and week 2 [days 8 and 11] of each cycle), oral thalidomide (100 mg daily in all cycles), and oral or intravenous dexamethasone (40 mg on days 1, 2, 8, 9, 15, 16, 22, and 23 of induction cycles 1 and 2 and days 1 and 2 of induction cycles 3 and 4 and 20 mg on days 8, 9, 15, and 16 of induction cycles 3 and 4 and days 1, 2, 8, 9, 15, and 16 of both consolidation cycles). Daratumumab was administered intravenously at a dose of 16 mg/kg of bodyweight once weekly in induction cycles 1 and 2 and once every 2 weeks during induction cycles 3 and 4 and consolidation. |
| (Moreau 2021) | 127 | Transplant-Eligible NDMM | Maintenance therapy | Daratumumab vs. Observation only | 16 mg/kg of daratumumab intravenously once every 8 weeks, up to a maximum of 2 years or observation only. |
| Mateos 2018 | 98 | Transplant-Ineligible NDMM | Induction therapy | Daratumumab-VMP vs. VMP | Intravenous daratumumab at a dose of 16 mg per kilogram of body weight was administered with oral or intravenous dexamethasone (to manage infusion reactions) at a dose of 20 mg once weekly in cycle 1, every 3 weeks in cycles 2 through 9, and every 4 weeks thereafter until disease progression or unacceptable toxic effects. Dexamethasone at a dose of 20 mg was substituted for prednisone on day 1 of each cycle.All the patients received up to nine (42-day) cycles of subcutaneous bortezomib (1.3 mg per square meter of body-surface area, twice weekly on weeks 1, 2, 4, and 5 of cycle 1 and once weekly on weeks 1, 2, 4, and 5 of cycles 2 through 9), oral melphalan (9 mg per square meter, once daily on days 1 through 4 of each cycle), and oral prednisone (60 mg per square meter, once daily on days 1 through 4 of each cycle). |
| Usmani 2021 (Usmani 2022) | 100 | Transplant-Eligible NDMM | Induction therapy | Elotuzumab-RVd vs. RVd | **With or without** elotuzumab at the dose determined during the phase 1 portion (10 mg/kg intravenously on days 1, 8, and 15 for cycles 1-2 and days 1 and 11 for cycles 3-8). And bortezomib (1.3 mg/m2 subcutaneously or intravenously on days 1, 4, 8, and 11), lenalidomide (25 mg orally on days 1-14), and dexamethasone (20 mg orally on days 1, 2, 4, 5, 8, 9, 11, and 12). |
| Voorhees 2020 | 30 | Transplant-Eligible NDMM | Induction therapy | Daratumumab-VRd vs. VRd | All patients received four 21-day induction cycles and two 21-day consolidation cycles of oral lenalidomide (25 mg daily on days 1-14), subcutaneous bortezomib (1.3 mg/m^2^ on days 1, 4, 8, and 11), and oral dexamethasone (20 mg on days 1, 2, 8,9, 15, and 16). Patients in the D-RVd group received IV daratumumab (16 mg/kg) on days 1, 8, and 15 of cycles 1 through 4 and day 1 of post-ASCT consolidation cycles (cycles 5 and 6). |
| Yong 2023 | 41 | Transplant-Eligible NDMM | Maintenance therapy | KCd vs. HSCT | **KCd:** KCd consolidation followed by carfilzomib maintenance. |
|  |  |  |  |  | HSCT: High-dose melphalan and autologous HSCT followed by carfilzomib maintenance. |
| Notes. 1. Post-treatment best-corrected visual acuity (BCVA) or visual acuity (VA) in letters. 2. Post-treatment central retinal thickness (CRT), including CMT, CFT and CST. 3. *Conversion formula: Letters = 85–50 × logMAR; †Median (IQR): median (interquartile); ‡Author country. 4. The T&E regimen is currently the commonly used treatment regimen for wet AMD in ophthalmology in Europe and the United States, and it continues to extend the follow-up and treatment interval by means of active defence, in the hope of achieving better treatment outcomes and reducing patient burden. The PRN regimen is currently the most commonly used treatment regimen in China, and it uses an initial composite phase treatment regimen with monthly follow-up for on-demand treatment. 5. Studies reporting VA: Kucuk 2019, Hanhart 2017, Hunt 2021. 6. Studies reporting on CFT: Kabatas 2020, Kaldirim 2018, Kucuk 2019; studies reporting on CST: Hunt 2021; studies reporting on CMT: Hanhart 2017 | | | | | |
